# Liver Transcriptome Analysis of the Black Porgy (*Acanthopagrus schlegelii*) under Acute Low-Temperature Stress

**DOI:** 10.3390/life13030721

**Published:** 2023-03-07

**Authors:** Yue Wang, Ziqiang Chen, Mingliang Wei, Zhijie Lin, Mingjun Shen, Fei Zhu, Chaofeng Jia, Qian Meng, Dafeng Xu, Shuran Du, Yanli Liu, Shuyin Chen, Caojin Zhang, Zhiyong Zhang, Zhiwei Zhang

**Affiliations:** 1 National Demonstration Center for Experimental Fisheries Science Education, Shanghai Ocean University, Shanghai 201306, China; 2 Jiangsu Marine Fishery Research Institute, Nantong 226007, China

**Keywords:** transcriptome, black porgy and low-temperature stress

## Abstract

High nutritional value and the development of efficient biotechnological methods of controlled production have made black porgy (*Acanthopagrus schlegelii*) an economically important fish in Chinese aquaculture in recent years. However, aquaculture production of the species faces multiple issues associated with reduced growth rate, low reproduction ability, and high mortality during production, which are associated with the species’ limited tolerance to low temperatures. To date, comprehensive information on the genetic-based mechanisms of cold tolerance and adaptation to low temperature in the species are still unavailable. In this study, the HiSeq™2500 (Illumina) sequencing platform was used to analyze the transcriptomic profile of the liver tissue in the black porgy subjected to different extents of cold shock, including a control temperature group (AS, T = 15 °C), an intermediate temperature group (AL1, T = 10 °C), and an acute low-temperature stress group (AL2, T = 5 °C). For this purpose, three standardized cDNA libraries of AS, AL1, and AL2 were established. We obtained 43,258,908, 48,239,072, and 38,983,833 clean reads from the AS group, AL1 group, and AL2 group, respectively. After pairwise comparison, 70 differentially expressed genes (DEGs) were identified in the examined fish groups. Among them, 60 genes were found to be significantly differentially expressed after trend analysis. GO annotation and enrichment results showed that they were mainly enriched into three categories: biological processes (12 subcategories), molecular functions (7 subcategories), and cellular components (7 subcategories). KEGG analysis results indicated that all significantly differentially expressed genes were annotated to 102 signaling pathways, including biological rhythm, cholesterol metabolism, glycerolipid metabolism, animal autophagy, FoxO signaling pathway, steroid biosynthesis, and regulation of adipocyte lipolysis and apoptosis. Four of them, namely: G6PC, GPX1, GCK, and HSPE1 were randomly selected for further qRT-PCR verification of data reliability obtained by RNA-Seq technology. In this study, we found that environmental acute cold stress mainly affected the black porgy’s biological processes related to metabolism, apoptosis, and signal transduction. The data that we have reported provides baseline information for further studies concerning the genetic responses of the black porgy under cold stress conditions, the improvement of its aquaculture production, and other economically important matters regarding their limited tolerance to cold shock.

## 1. Introduction

Water temperature greatly affects the physiology and behavior of animals, including fish [1]. As cold-blooded animals, fish are able to adapt themselves to temperature changes by adjusting their own metabolism rate [2]. However, this can only take place within a tolerable range as a too-low or too-high temperature can cause lethal and irreversible physiological disruptions. Both chronic and acute low-temperature stress may have a lethal effect on the fish’s immune and metabolic system caused by detrimental changes in the fluidity and stability of cell membranes, bringing huge economic losses under aquaculture production [3]. Black porgy (*Acanthopagrus schlegelii)* is a representative of the Sparidae family of fishes (Order: Perciformes), which natively inhabits the bottom of shallow seas across the western Pacific; from North-Eastern Russia to the middle coast of Vietnam [4]. It is a predatory fish species that usually attains 50 cm in length and a body mass of up to 3 kg. High taste and nutritional values, as well as strong resistance to diseases and changing environmental conditions, have made the species an economically important subject of aquaculture production in Southeast Asian countries [5]. However, available observations indicate that when water temperature drops below 5 °C, the metabolic rate of the black porgy becomes critically impaired, which is manifested by reduced swimming and food intake abilities [6]. Because of these limited adaptation abilities to low temperatures, the species is mainly produced in Japan, South Korea, North Korea, Vietnam, and southern China [7]. The available studies indicate that low-temperature stress hinders the species’ growth performance but the information on potential genetic-based mechanisms responsible for this process remains unclear. Multiple genes may be involved in the regulation of this pathway, which needs to be further studied.

Transcriptome analysis is a powerful tool for studying biological development, physiological stress, as well as immune and metabolic capacity. To this date, researchers have described transcriptome changes in many fish under cold stress conditions, including the *Takifugu fasciatus* [8], catfish [9], tilapia [10], silver pomfret [11], grass carp [12], and the gilthead sea bream [2]. These studies have identified a large number of cold-regulated genes associated with daily and seasonal low-temperature changes. However, although black porgy is one of the most popular economic marine species, transcriptome analysis has not yet been used to study the genetic-based response mechanisms in the species under acute low-temperature stress.

The purpose of this study was to investigate the molecular changes in the black porgy when subjected to acute low-temperature stress and to find out the related regulatory genes taking part in the pathways responsible for cold stress in the species. The liver, which has the functions of detoxification, immune defense, hormone secretion, and synthesis [13], is one of the most important metabolic organs in fish. In this paper, the transcriptome libraries of black porgy liver under control temperature (AS, T = 15 °C), intermediate temperature (AL1, T = 10 °C), and stress temperature (AL2, T = 5 °C) were constructed by the HiSeq^TM^2500 (Illumina, San Diego, CA, USA) sequencing platform. The result of this study can help us to understand the mechanism of the low-temperature tolerance of black porgy in different environments in order to cope with the large number of deaths caused by cold waves while laying a foundation for us to improve the effectiveness of its aquaculture production under low-temperature conditions.

## 2. Materials and Methods

### 2.1. Experimental Design and Sample Preparation

All the experimental procedures described in this paper follow the international standards on animal welfare and were approved by a local ethic committee.

A total of 200 healthy, 1-year-old black porgy juveniles (105.31 ± 13.15 g, 15.5 ± 1.4 cm) were collected from Jiangsu Province Black Sea-Bream Breeding Farm located in Nantong city and kept in a recirculated rectangular tank of about 9 m^3^ (3.9 m × 2.6 m × 0.9 m) with a water exchange rate of 50 percent every day. Fish were kept in 15 °C water with a salinity of 21–22‰, within a volume of 3000 L, and with an oxygen concentration above 6 mg/L. Fish were fed by commercial pellet feed twice a day, i.e., at 9:00 a.m. and 6:00 p.m., an amount of 3% of their biomass. After two weeks of rearing, the feeding was stopped for 24 h in-tank, followed by an acclimation period of 48 h in foam boxes before the start of the experiment. Because 15 °C is the optimum temperature for the physiology and the metabolism of black porgy, it was used as a control temperature (AS). On the other hand, the temperature of 5 °C is known to be a lower lethal bound for the species, thus, this was used as an acute low-temperature for the stress group (AL2). Finally, a temperature of 10 °C was used as an intermediate between the optimal and lethal temperature (group AL1). After the acclimation period, a total of 90 fish were randomly selected and placed in 9 foam boxes of around 11.5 L volume (28.5 cm × 22.5 cm × 18.0 cm). Each experimental group was kept in three separate foam boxes (triplicates) of 10 fish each under identical conditions as described above to mitigate the influence of any possible factors other than the temperature on the transcriptome profile of the examined fish. For the AL1 and AL2 groups, the temperature was decreased by a rate of 2.5 °C per hour to avoid a lethal temperature change effect on the fish. After the temperature was set, the fish were kept in the boxes for 24 h and all fish were then anesthetized in tricaine mesylate solution (300 mg/L; MS-222) and sacrificed. Immediately after collection, the liver tissues were put into separate tubes and stored at −80 °C until further analysis.

### 2.2. Preparation for RNA-Seq Libraries and Sequencing

Total RNAs from liver tissues were isolated using a Trizol reagent kit (Invitrogen, Carlsbad, CA, USA). The RNA integrity and DNA contamination were detected by RNase-free 1% agarose gel electrophoresis with a constant voltage of 120 V, 1× TAE denaturation buffer, and 0.5 μg/mL EB. The concentration and purity of RNA were measured using a NanoPhotometer spectrophotometer (Implen NanoPhotometer, Westlake Village, CA, USA; OD260/280:1.8–2.4). Agilent 2100 Bioanalyzer (Agilent Technologies, Santa Clara, CA, USA) was used to assess RNA quality. Samples with RNA integrity number (RIN) > 7.0 were subjected to RNA-Seq sequencing.

According to the manufacturer’s instructions, 1μg of total RNA per pool was reverse transcribed using the M-MuLV kit (Sangon Biotech, Shanghai, China). Double-strand of cDNA was synthesized by PCR amplification and then used for library preparation. Resulted cDNA was then purified using the QiaQuick PCR Extraction Kit (Qiagen, Venlo, The Netherlands), end-repaired, poly(A) added, and ligated to Illumina sequencing adapters. The library was then sequenced on the Illumina HiSeq2500 sequencing platform after quality inspection.

### 2.3. Assembly and Annotation

Adapters and low-quality sequences (with a ratio of low-quality bases of more than 50% and a ratio of ‘N’ above 10%) were removed using the Fastp toolkit (HaploX Biotechnology, Shenzhen, China). Sequences of rRNA were mapped and removed by Botwie2 software 2.24 (Langmead & Salzberg University of Georgia, Athens, GA, USA) [14] and only effective reads were retained for subsequent transcriptome analysis. An index of the reference genome was built using reference data on the black porgy genome reported by Zhang [13]. For this purpose, paired-end clean reads were mapped to the reference genome using HISAT2.2.4 with “RNA-strandness RF”, setting all other parameters as a default.

### 2.4. Sample Relation Analysis

Principal component analysis (PCA) is a powerful technique for revealing the relationship between samples. We used the R software version 2.13 (Robert Gentleman&Ross Ihaka, University of Auckland, Auckland, New Zealand) package gmodels (http://www.rproject.org/, accessed on 31 March 2021) for PCA, and reduced the dimension of the original data by variance decomposition so as to convert multiple indicators into a few comprehensive indicators. The correlation of the samples was judged by the distance between the points in the PCA diagram, which indicated closely related samples when clustered on the data plot.

### 2.5. Differentially Expressed Genes and Enrichment Analysis

Differentially expressed genes (DEGs) between groups (AS vs. AL1, AS vs. AL2, and AL1 vs. AL2) were analyzed using the DESeq2 software version2.0. The resulting *p*-value was corrected using the accepted Benjamini–Hochberg method to control the false discovery rate (FDR). After adjustment, genes with an adjusted *p*-value < 0.05 and a fold-change ≥ 2 were assigned as DEGs. After that, the selected DEGs were used for trend analysis by Short Time-series Expression Miner software (Version 1.1 Jason Ernst and Ziv Bar-Joseph Cainegie Mellon University, Pittsburgh, PA, USA) (http://www.cs.cmu.edu/~jernst/stem, accessed on 1 January 2005) to screen out the biological characteristics of some significantly differentially expressed genes (such as the continuous increase in expression). Then, Gene Ontology (GO) and Kyoto Encyclopedia of Genes and Genomes (KEGG) functional enrichment analysis was performed on the genes with significant differences in the trend. Firstly, the significantly different genes were mapped to each term (Pathway) of the GO (KEGG) database (http://www.geneontology.org/), and the number of genes in each term (Pathway) was calculated. Then, the *p*-value was calculated by hypothesis test. The obtained *p*-value was corrected by FDR and Q-value ≤ 0.05 was used as the threshold. The GO term and Pathway that satisfied this condition were defined as GO term and Pathway with significant enrichment of differential genes.

### 2.6. Validation Using Real-Time Quantitative PCR

We randomly selected four candidate genes (G6PC, GCK, GPX1, and HSPE1) for qRT-PCR validation. Total RNA was extracted from the liver tissue of the control and experimental groups by the Trizol method (Invitrogen). First-strand of cDNA was synthesized from 1 μg total RNA using an M-MuLV First Strand cDNA Synthesis Kit (Sangon Biotech, Shanghai, China) according to the manufacturer’s instructions. Obtained cDNA were used as a template and β-actin was set as a housekeeping gene and qPCR assays were performed for each candidate gene on samples originating from all experimental groups. Allele ID 6.0 software (http://www.premierbiosoft.com/) was used to design specific primers for all examined candidate genes based on their sequences acquired after transcriptome sequencing (Table 1). After qPCR reaction optimization amplification, efficacy for all genes varied between 91–95% (including the housekeeping gene). qPCR amplifications were performed in triplicates, including their negative controls, using Tiangen’s SuperReal PreMix Plus (SYBR Green) reagent (Beijing, China). Three-step RT-PCR program was performed as follows: 95 °C for 15 min, followed by 40 cycles of 95 °C for 10 s, 55 °C for 31 s, and 72 °C for 32 s. The measurements of relative gene expression were performed by the 2^−ΔΔCt^ method implemented in an ABI 7300 plus Real-Time PCR quantitative PCR machine (Applied Biosystems, Foster City, CA, USA).

## 3. Results

### 3.1. Transcriptome Sequencing and Assembly

The raw data of transcriptome sequencing were deposited in NCBI’s Short Read Archive (SRA) database, under accession number PRJNA680912. Transcriptome sequencing provided 43,332,655, 48,341,663, and 39,050,327 raw reads from the liver of AS, AL1, and AL2 groups, respectively. The share of clean reads was higher than 99.76% in each experimental group (Table 2). HISAT2 software was used to match the assembled sequencing data with the available database genome of the black porgy. The matching rate of 80.24–84.67% indicates that the assembled results of the transcriptome data are reliable.

A total of 19,465 genes were annotated in the black porgy reference genome which was published in 2018 [14], while 14,009, 13,891, and 14,009 genes were annotated in the AS, AL1, and AL2 groups, respectively. A total of 1064 new genes were annotated in the black porgy genome under cold stress, while 1005, 1004, and 1012 new genes were annotated in the AS, AL1, and AL2 groups, respectively. A total of 20,529 genes were annotated in the black porgy genome, while 15,014, 14,895, and 15,021 genes were annotated in the AS, AL1, and AL2 groups, respectively (Table 3).

### 3.2. Sample Relationship

**Figure 1:**
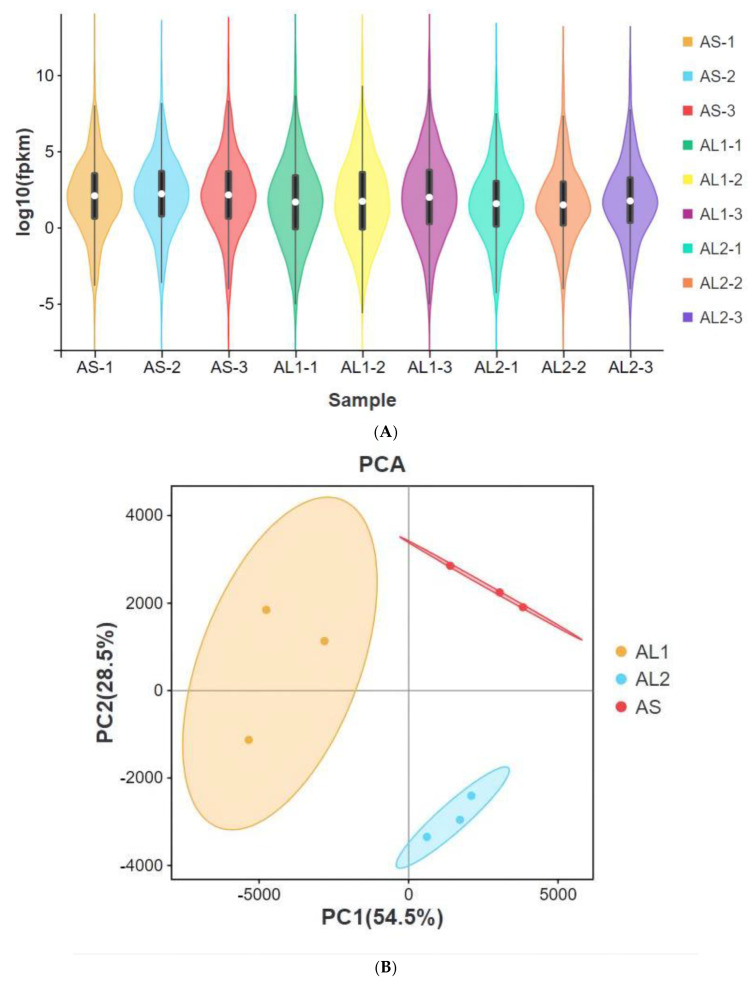
Violin plot of 3609 differentially expressed genes (DEGs) among examined groups of the black porgy exposed to different temperatures (**A**). Principal component analysis of 3609 DEGs (**B**). Note: AS represents the 15 °C control group, AL1 represents the 10 °C intermediate temperature stress group, and AL2 represents the 5 °C low-temperature stress group.

A total of 3609 differentially expressed genes (DEGs) (FDR < 0.05, |log2 (fold-change)| ≥ 1) were obtained after the transcriptome sequencing of black porgy liver tissue. Expression patterns of 3609 DEGs in different samples were presented on a violin plot (Figure 1A). The obtained results on whole transcriptome analysis indicated differences in the expression pattern in the black porgy when exposed to different temperatures. Then, principal component analysis (PCA) considered the expression levels of 3609 DEGs and reduced them to a plane. We can see from the PCA plot (Figure 1B) that the contribution of the first principal component (PC1) to the sample difference was 54.5%. In addition, the contribution of the second principal component (PC2) to the sample difference was 28.5%. Through analysis, it can be seen that black porgy show the characteristic of intergroup separation and intragroup aggregation at different temperatures. This shows that the repeatability of the sample is good enough for the experiment.

### 3.3. Basic Characteristics of Identified Differentially Expressed Genes (DEGs)

**Figure 2:**
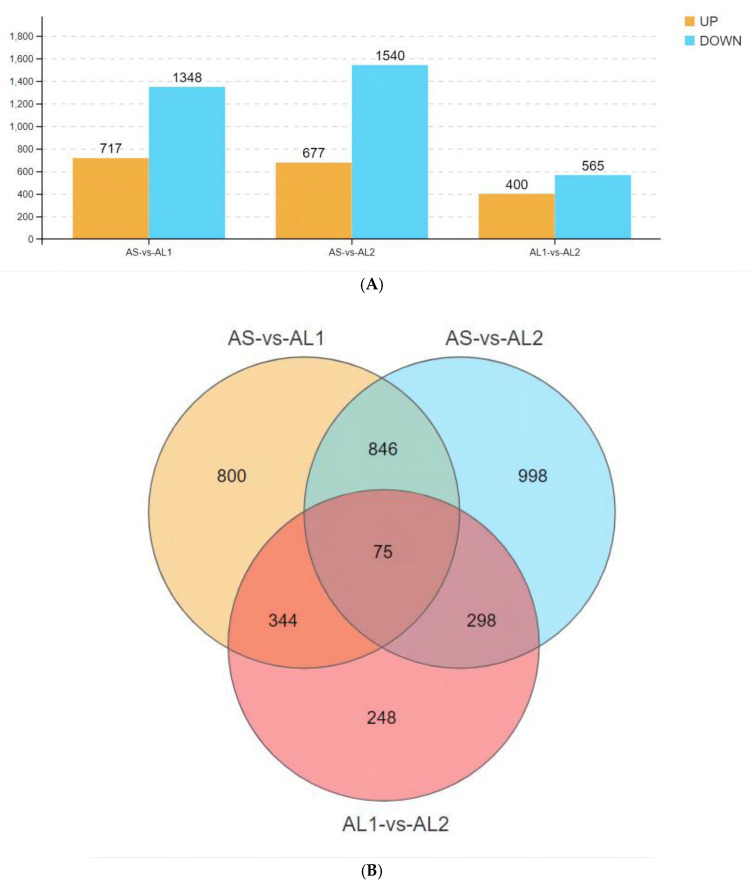
The histogram of differentially expressed genes between each pair of examined groups (**A**). Wayne diagram of differentially expressed genes between all examined groups (**B**). Note: AS represents the 15 °C control group, AL1 represents the 10 °C intermediate temperature group, and AL2 represents the 5 °C low-temperature stress group. The orange color represents differentially up-regulated genes and the blue color represents differentially down-regulated genes (Q < 0.05).

All the 3609 differentially expressed genes were subjected to pairwise analysis between all examined fish groups. In AS vs. AL1 comparison, a total of 2065 DEGs were identified, where 717 were up-regulated and 1348 were down-regulated. In AS vs. AL2 comparison, 2217 DEGs were identified where 677 were up-regulated and 1540 down-regulated. Finally, in the AL1 vs. AL2 comparison, 965 DEGs were identified of which 400 were up-regulated and 565 down-regulated (Figure 2A). A total of 75 differentially co-expressed genes were found among the 1565 crossover DEGs obtained by overall comparison of AS, AL1, and AL2 groups (Figure 2B).

### 3.4. Trend Analysis of Differentially Expressed Genes

**Figure 3:**
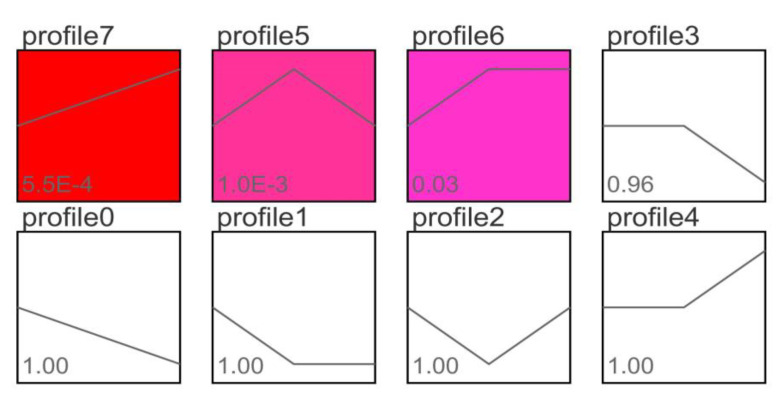
Trend analysis of 75 differentially co-expressed genes. Note: Each small square represents a profile and the line in each profile represents an expression trend of all genes in the profile. Each inflection point group is a set of gradient data. The leftmost side of the square is the 15 °C control group (AS), the middle is the 10 °C stress group (AL1), and the rightmost is the 5 °C stress group (AL2). The value in the lower left corner of each trend box represents the significance of the differential genes enriched in the trend module (by comparing the number of genes enriched to a certain trend with the random distribution expectation, according to the set significance threshold, when *p* < 0.01 it shows an extremely significant difference when *p* < 0.05 it shows a significant difference). The trend with color labeling is significant enrichment (color depth represents the level of significance), and the trend without color labeling is not significant. The top title of each small square is the number of the profile.

After trend analysis of 75 differentially co-expressed genes among all examined groups, 8 different trend profiles were obtained as shown in Figure 3. In these 8 different trend files, according to the significant value shown at the lower left corner of each profile box, the genes in profile 7, profile 5, and profile 6 were shown to be significantly different after trend analysis. Furthermore, the genes in the remaining 5 profiles were non-significantly different. Among them, the expression trend of 24 extremely significant DEGs (such as diablo, Tis11, myc, ARL14, BHLHE40, Tnfsf10, CYP27 B1, Nr1d2, Vegfa, GADD45 G, nfi13, junb, Irs2, ddit4, Trib1, PRELID3B, Soat1, and adrb2) in profile 7 continued to increase in AL1 to AL2. In profile 5, the expression levels of 24 extremely significant DEGs (such as Sqstm1, rab4b, RELB, U1k2, foxo1a, LTBR, ZFYVE1, Tat, VPS37B, TNIP2, EIF4E, MTERF2, SLC4A4, and GDF15) in AS to AL1 showed an upward trend, and the expression levels in AL1 to AL2 showed a downward trend. In profile 6, the expression levels of 12 significant DEGs (such as chac1, KLF9, Zfand5, LPL, CIART, and Pnpla2) in AS to AL1 showed an upward trend, and the expression levels in AL1 to AL2 remained unchanged. The relationship between the genes in profiles 7, 5, and 6, and the expression levels of each group, is shown in Supplementary Table S1. In summary, after trend analysis of 75 differentially co-expressed genes, there were 60 significant DEGs and 15 non-significant DEGs.

### 3.5. GO and KEGG Enrichment Analysis

**Figure 4:**
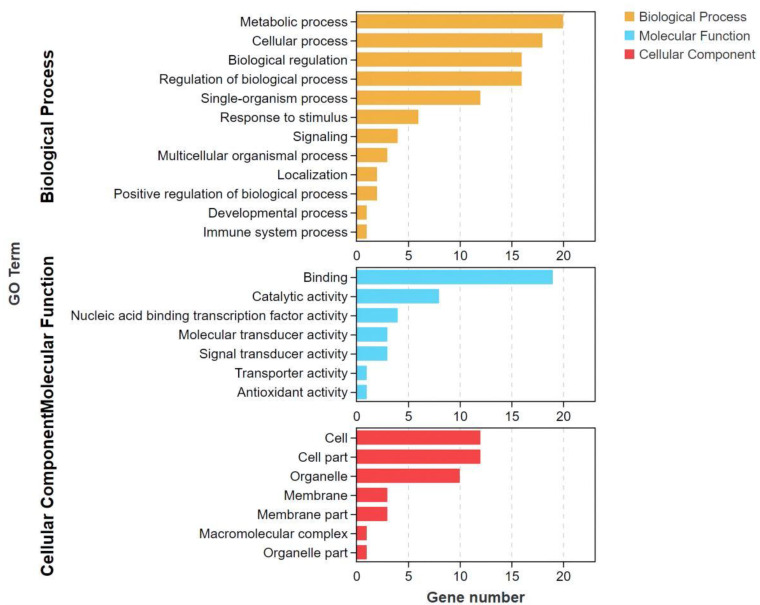
GO enrichment bar graph.

A total of 60 significantly different among all examined fish groups’ co-expressed genes obtained by trend analysis were enriched to 26 GO terms (Supplementary Table S2). The enrichment results were divided into three categories, namely: biological process (BP), metabolic function (MF), and cellular component (CC) (Figure 4). In BP, differential genes were mainly enriched in metabolic processes, cellular processes, biological regulation, and biological regulation processes. For MF, the differential genes were mainly enriched in binding, followed by catalytic activity. In CC, it was mainly enriched in cells, cell parts, and organelles. In general, most of the DEGs were enriched by terms belonging to BP.

**Figure 5:**
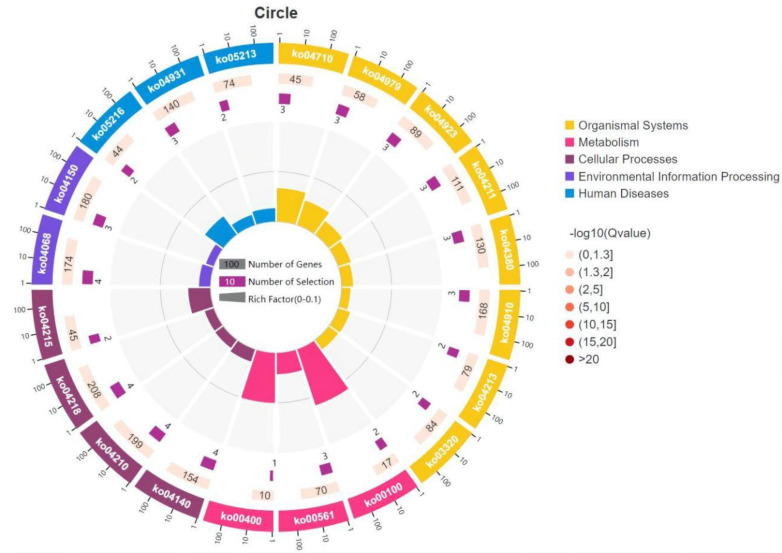
Enrichment circle map showing 20 most annotated pathways to 60 significantly differential genes in KEGG analysis. Note: The first circle represents enrichment classification, different colors represent different classifications, and outside the circle is the scale of gene number; The second circle represents the number of the classification and the Q value in the background gene, the length of the bar indicates the number of genes, the color depth indicates the size of the Q value, the deeper the color, the greater the possibility of enrichment; The third circle represents the number of differential genes enriched in this classification; The fourth circle represents the Rich Factor value of each classification (the ratio of differential genes to the total number of classified genes in this classification).

**Figure 6:**
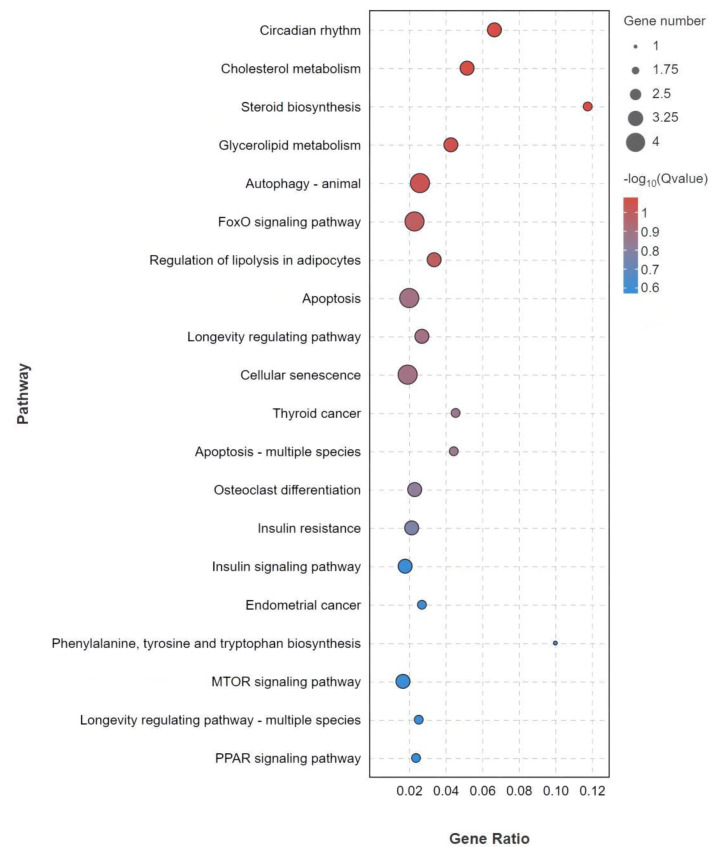
Bubble plot of 20 most annotated pathways to 60 differentially expressed genes.

In this study, 60 significantly different genes were annotated by KEGG analysis to 102 signaling pathways. Among them, 20 of the most annotated pathways, together with their genes, are shown in Supplementary Table S3. The most enriched 22 differential genes and 14 differential genes were related to organic system and cellular process pathways, respectively (Figure 5). KEGG analysis identified three pathways involved in human disease, including thyroid cancer (ko05216), insulin resistance (ko04931), and endometrial cancer (ko05213). Figure 6 shows the top 20 signaling pathways enriched by 60 significantly different genes. The most annotated pathways were circadian rhythm, cholesterol metabolism, glycerolipid metabolism, animal autophagy, FOXO signaling pathway, steroid biosynthesis, regulation of lipolysis in adipocytes, and apoptosis.

### 3.6. Validation of RNA-Seq Data by qRT-PCR

**Figure 7:**
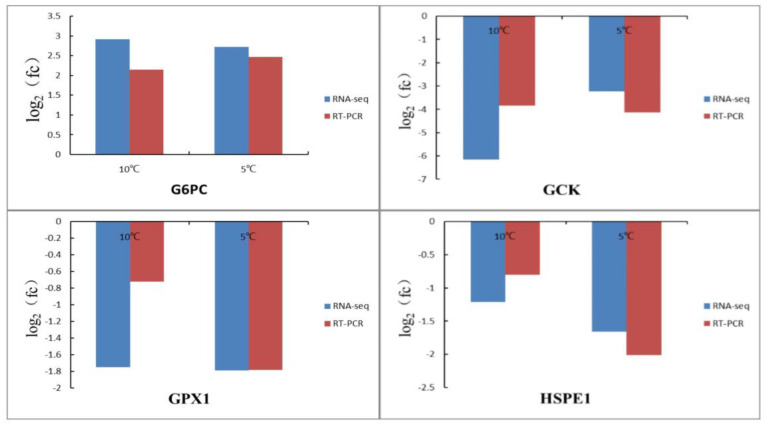
Validation of DEGs in black porgy under low temperature by RT-PCR. Note: Blue pillars indicate the result of RNA-Seq, and red pillars indicate the result of RT-PCR.

In order to verify the reliability of RNA-Seq sequencing data, four differentially expressed genes (G6PC, GPX1, GCK, and HSPE1) related to low-temperature stress were randomly selected for qPCR analysis (Figure 7). In both qPCR and RNA-Seq analysis, the expression trends were basically the same. However, the expression levels of examined genes were different. These results indicate that the data produced by RNA-Seq technology is reliable.

## 4. Discussion

In order to get a low-temperature tolerance variety of black porgy, it is necessary for us to explore the molecular mechanism under cold stress. RNA-Seq sequencing technology provides a low-cost, highly repeatable, and powerful tool for studying the transcription level of specific tissues or cells at a certain period [15]. In this study, the transcriptome sequencing technique was used to analyze the transcriptome profile of black porgy liver tissue under acute low-temperature stress. Seventy-five differentially co-expressed genes were obtained by pairwise comparison among AS, AL1, and AL2 groups. Then, after trend analysis, 60 significant DEGs were analyzed. Finally, GO and KEGG enrichment analysis was performed on those 60 significant DEGs. The results of GO enrichment analysis showed that these DEGs were mainly related to metabolic processes, cellular processes, biological regulation processes, cell parts, and binding. In turn, the results of enrichment analysis showed that these DEGs were mainly related to biological rhythm, cholesterol metabolism, glycerolipid metabolism, animal autophagy, FOXO signaling pathway, steroid biosynthesis, regulation of adipocyte lipolysis, and apoptosis. GO enrichment analysis mainly enriches some processes in which KEGG focuses on the pathway. Thereafter, the reliability of the RNA-Seq data was verified by qPCR. For this purpose, four DEGs were selected to examine the relationship between qPCR and RNA-Seq data expression levels. The results showed that qPCR and RNA-Seq expression trends were basically the same. However, the expression levels of both examined genes were different. These results indicate that the RNA-Seq sequencing data is reliable.

In this study, fewer immune pathways were enriched while more were related to apoptosis and cell senescence pathways in black porgy. This may be due to the body’s immune system not being strong enough to resist external acute low-temperature stress. If external stress exceeds the body’s tolerance, then the cell eventually goes to the apoptotic pathway. Relevant research has been reported in fish [16], shrimp [17,18], and crabs [19,20] that the body responds to external environmental stresses by regulating its own immune system. Heat shock protein (HSP) are a highly conserved protein family that maintain cellular homeostasis and play an important role in the early stage of the low-temperature-tolerance mechanism in fish [21,22]. The up-regulation of heat shock protein levels can increase the level of peroxidase, thereby scavenging excessive free radicals produced by stress in fish [23]. Therefore, HSP can be used as a detection index of cell function in the process of the stress response. The HSPE1 gene detected in this study showed a down-regulated expression trend. It can be speculated that the black porgy activated its own immune system in order to resist external stress under acute low-temperature stress. However, the expression of the HSPE1 gene may be inhibited due to the persistence of stress. Otherwise, HSP genes may be activated in response to high-temperature shock. Glutathione-S-transferase (GST) is a detoxification-related protein that promotes the binding of tripeptide GSH to some harmful electrophilic substrates [24]. GST has the function of scavenging ROS and enhancing detoxification, thus preventing cell membrane and other macromolecular damage [25]. Therefore, we can improve the tolerance of the body to cold stress by promoting the synthesis of GST and the high expression of the HSP protein.

The liver is an important organ of fish metabolism that is involved in protein synthesis, detoxification, lipid metabolism, and essential amino acid metabolism [26]. It mainly stores glycogen from the gastrointestinal tract to maintain blood glucose balance. It has been reported in Yellow drum [27] that energy is mainly provided by the consumption of cholesterol, triglyceride, and glucose in the early stage of cold stress and by the utilization of protein and fat metabolism in the late stage of cold stress. It can be inferred from the KEGG enrichment pathway in this study that black porgy mainly provide energy for the body through cholesterol metabolism, steroid biosynthesis, and lipid metabolism under acute low-temperature stress. However, the energy provided to the fish through metabolism may be used more to respond to low-temperature stress and only a small amount was used for protein synthesis to promote growth. Therefore, once the external temperature is beyond the tolerance range of the fish, the body cells will be in a state of apoptosis. In addition, low-temperature stress can destroy the fluidity and stability of the cell membrane. The physical properties of the phosphate membrane in the cell membrane mainly depend on the saturation of its fatty acid composition [28]. The synthesis of fatty acids is related to acetyl coenzyme A synthase (AACS), which is responsible for the synthesis of cholesterol and fatty acids from the ketone in adipose tissue [29]. Therefore, we can synthesize cholesterol and fatty acids by promoting the synthesis of AACS so as to improve the body ‘s metabolic capacity in response to low-temperature stress. 

**Figure 8:**
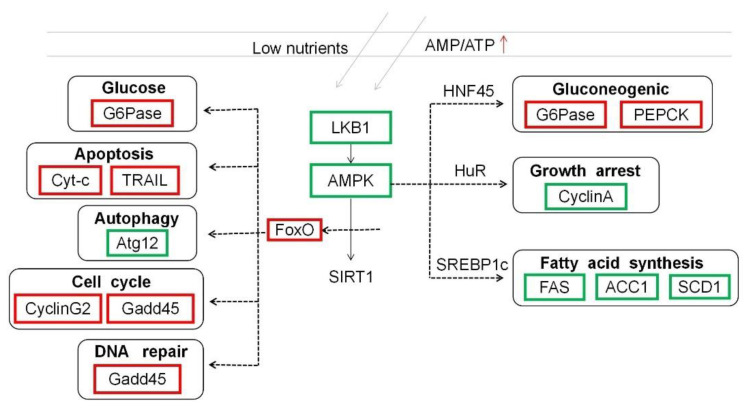
AMPK and FOXO signaling pathway.

Signal transduction in organisms is essential for the body to adapt to environmental stress. However, there is still a lack of information on the signal transduction mechanism of teleost fish under low-temperature stress. It has been reported [30] that when the body is under external stress, the adenosine monophosphate-activated protein kinase (AMPK) signaling pathway is activated in order to maintain the normal energy metabolism of cells. AMPK mainly provides energy for the body to cope with environmental stress by regulating protein synthesis, fat metabolism, and glycogen synthesis. In this experiment, black porgy needed to consume more energy to maintain the metabolic balance under acute low-temperature stress. Therefore, less energy was used for protein and glycogen synthesis, which caused the late AMPK signal transduction pathway to block, showing a downward trend (Figure 8). The down-regulated expression of the AMPK signal transduction pathway blocked the fatty acid synthesis pathway. Furthermore, the expression of fatty acid synthase (FAS) [31], acetyl-CoA carboxylase-1 (ACC1) [32], and stearoyl-CoA dehydrogenase-1 (SCD1) [33], which are candidate factors of fat deposition, were down-regulated. However, sterol regulatory element binding protein (SREBP1C) is an important nuclear transcription factor [34]. It can bind to FAS, ACC1, and SCD1 on the sterol response element sequence, thereby activating the transcription of fatty acid de novo synthesis-related enzyme gene and affecting lipid production. Therefore, the SREBP1C transcription factor was affected by the down-regulation of the AMPK signaling pathway, which reduced the synthesis rate of fatty acids and affected the normal metabolism of the body. As a downstream pathway of the AMPK signaling pathway, FOXO1 plays an important role in maintaining intracellular glucose metabolism, regulating the cell cycle, DNA repair, and animal autophagy [35]. The activation of the FOXO signaling pathway increased blood glucose levels, thereby up-regulating glucose-6-phosphatase (G6Pase) and activating the gluconeogenesis pathway. This may be due to the conversion of non-sugar substances into sugars to provide energy for the body through gluconeogenesis under acute low-temperature stress. This result is consistent with the signal transduction in the liver tissue of tilapia under low-temperature stress [10]. In addition, FOXO has also shown the effect on regulating myoblast apoptosis and differentiation which was confirmed in fish muscle cells [35]. Growth arrest and DNA damage repair gene 45 (Gadd45) is an important DNA damage repair gene that regulates cellular immunity and apoptosis [36]. In this study, FOXO expression was up-regulated so the Gadd45 gene was up-regulated to control the cell cycle and repair DNA damage. At the same time, it also activated the expression of tumor necrosis factor-related apoptosis-inducing ligand (TRAIL) and cytochrome C (Cyt-c) in the apoptotic pathway. This result may damage the genetic mechanism in black porgy during low-temperature stress. If the stress cannot be stopped, the body will eventually move toward the apoptotic pathway.

In summary, by promoting the synthesis of GST and the high expression of HSP, black porgy’s tolerance to low-temperature stress can be improved. This can also synthesize cholesterol and fatty acids by promoting the expression of AACS to provide energy for the body under stress. FAS, ACC1, and SCD1 can be used as candidate factors for fat deposition. When the body is in a stressful environment, the metabolic level of the body can be improved by up-regulating the expression of the three enzyme genes above.

## 5. Conclusions

In this study, the 60 DEGs in black porgy under low-temperature stress were mainly enriched in metabolism, apoptosis, and signal transduction pathways. Our results revealed that candidate fat deposition factors (FAS, ACC1, and SCD1) and DNA damage repair gene (Gadd45) are important response factors in temperature stress. This research explored the regulation network and the molecular mechanisms in black porgy under cold stress. Those results will be useful for the species’ safe overwintering outdoors in the northern part of China, which will reduce its aquaculture cost and increase the benefit. This might also provide a basic theory for the selection of cold-tolerance variety in black porgy.

## Figures and Tables

**Table 1:** Genes and specific primers used for validation of RNA-Seq data by real-time PCR.

Gene Name	Sequence (5′–3′)	Amplification Efficiency/%
β-actin	F: CGACGGTCAGGTCATCAC	91.957
	R: GCCAGCAGACTCCATTCC	
G6PC	F: TACACCGAGACCAAGAAG	92.611
	R: GACAGCAGGTAGAAGAGG	
GCK	F: GACATCATCTCAAGCACAAG	94.953
	R: GGCAACCACATCCATCTC	
GPX1	F: ATCTGCCCTGATGACTGAC	93.368
	R: CCTGCTGTAACGCTTGAAG	
HSPE1	F: AGAAGGCTCAAGGCAAAG	93.504
	R: TCACGGAACAGGAAATAGTC	

**Table 2:** Statistical table of data filtering.

Sample	Rawdata	CleanData (%)	Adapter (%)	LowQuality (%)
AS-1	41,124,586	99.84	0.02	0.14
AS-2	44,151,136	99.81	0.02	0.16
AS-3	44,722,244	99.84	0.02	0.14
AL1-1	50,589,846	99.78	0.03	0.19
AL1-2	44,156,390	99.76	0.04	0.20
AL1-3	50,278,754	99.82	0.02	0.15
AL2-1	37,965,772	99.85	0.02	0.13
AL2-2	38,403,050	99.82	0.02	0.15
AL2-3	40,782,160	99.82	0.03	0.15

**Table 3:** Statistical table of gene comparison.

Sample	Refer Genes	Sequenced Refer Genes	Average Sequenced Refer Genes	Novel Genes	Sequenced Novel Genes	Average Sequenced Novel Genes	Total Genes	Sequenced Total Genes	Average Sequenced Total Genes
all	19,465	16,608		1064	1064		20,529	17,672	
AS-1	19,465	13,897	14,009	1064	999	1005	20,529	14,896	15,614
AS-2	19,465	14,070		1064	1011		20,529	15,081	
AS-3	19,465	14,060		1064	1004		20,529	15,064	
AL1-1	19,465	13,959	13,891	1064	1005	1004	20,529	14,964	14,895
AL1-2	19,465	13,602		1064	1001		20,529	14,603	
AL1-3	19,465	14,113		1064	1007		20,529	15,120	
AL2-1	19,465	13,905	14,009	1064	1003	1012	20,529	14,908	15,021
AL2-2	19,465	14,081		1064	1021		20,529	15,102	
AL2-3	19,465	14,041		1064	1011		20,529	15,052	

## Data Availability

We have upload the data to NCBI website.

## Supplementary Materials

The following supporting information can be downloaded at: https://www.mdpi.com/article/10.3390/life13030721/s1, Table S1: Expression of 60 DEGs in profile 5, profile 6 and profile 7 at different temperatures. Table S2: GO terms enriched from 60 significantly differentially expressed genes. Table S3: List of 20 most annotated pathways to 60 significantly differentially expressed genes identified in this study enriched in KEGG analysis.
