# Supplementary material for: Liver Transcriptome Analysis of the Black Porgy (Acanthopagrus schlegelii) under Acute Low-Temperature Stress
Source: Life (Basel). 2023 Mar 7;13(3):721. doi: 10.3390/life13030721 (PMC10057800; doi:10.3390/life13030721)
Supplement: Supplementary file 1 [file life-13-00721-s001.zip › life-2134126-supplementary.pdf]

Supplement Table S1. Expression of 60 DEGs in profile 5, profile 6 and profile 7 at different temperatures

ID	Profile	AS	AL1	AL2	Symbol
BP_GLEAN_10000233	7	0	2.8	4.97	diablo
BP_GLEAN_10002830	7	0	2.82	5.08	diablo
BP_GLEAN_10003165	7	0	1.79	3.27	Tis11
BP_GLEAN_10005396	7	0	1.21	2.41	myc
BP_GLEAN_10005639	7	0	1.75	4.05	ARL14
BP_GLEAN_10005816	7	0	1.1	2.73	BHLHE40
BP_GLEAN_10006143	7	0	1.12	2.36	Tnfsf10
BP_GLEAN_10007279	7	0	2.78	6.34	CYP27B1
BP_GLEAN_10007289	7	0	1.13	2.89	Nr1d2
BP_GLEAN_10007370	7	0	1.71	3.48	NR1D2
BP_GLEAN_10008929	7	0	1.95	3.68	Vegfa
BP_GLEAN_10011108	7	0	2.11	3.55	GADD45G
BP_GLEAN_10011604	7	0	2.78	3.8	nfil3
BP_GLEAN_10011616	7	0	1.19	3.61	junb
BP_GLEAN_10011841	7	0	2.14	3.5	Irs2
BP_GLEAN_10014551	7	0	2.88	4.01	ddit4
BP_GLEAN_10015535	7	0	1.75	2.97	Trib1
BP_GLEAN_10015671	7	0	2.16	3.29	PRELID3B
BP_GLEAN_10015689	7	0	1.1	2.64	SOAT1
BP_GLEAN_10015732	7	0	1.48	2.69	Errfi1
BP_GLEAN_10016005	7	0	1.32	3.63	Bhlhe40
BP_GLEAN_10018435	7	0	2.74	4.13	MSH5
BP_GLEAN_10019203	7	0	2.35	3.5	Cebpd
MSTRG.9053	7	0	2.79	4.56	adrb2
BP_GLEAN_10000555	5	0	3.65	1.14	Sqstm1
BP_GLEAN_10005592	5	0	2.36	1.07	rab4b
BP_GLEAN_10005601	5	0	2.55	1.15	RELB
BP_GLEAN_10006362	5	0	2.78	1.25	Ulk2
BP_GLEAN_10006563	5	0	3.83	1.89	foxo1a
BP_GLEAN_10006783	5	0	2.34	-1.35	LTBR
BP_GLEAN_10008121	5	0	2.69	1.23	ZFYVE1
BP_GLEAN_10009421	5	0	3.31	1.47	Tat
BP_GLEAN_10011321	5	0	2.64	1.07	VPS37B
BP_GLEAN_10012150	5	0	4.27	1.69	TNIP2
BP_GLEAN_10016747	5	0	1.36	-1.17	EIF4E
BP_GLEAN_10017349	5	0	2.86	1.35	MTERF2
BP_GLEAN_10017986	5	0	2.81	-1.14	SLC4A4
BP_GLEAN_10018132	5	0	2.91	-1.8	--
BP_GLEAN_10018743	5	0	1.27	-1.06	Homez
BP_GLEAN_10018847	5	0	2.69	1.31	PDE4B
BP_GLEAN_10018972	5	0	3.21	1.5	GDF15
MSTRG.13130	5	0	3.28	1.34	--
MSTRG.14166	5	0	3.17	1.3	--
MSTRG.14168	5	0	3.57	1.03	--
MSTRG.14645	5	0	1.98	-1.1	--
MSTRG.5848	5	0	3.51	1.39	--
MSTRG.8213	5	0	3.15	-1.96	--
MSTRG.8997	5	0	2.7	1.33	--
BP_GLEAN_10008119	6	0	5.24	2.91	chac1
BP_GLEAN_10010872	6	0	4.15	2.63	--
BP_GLEAN_10012428	6	0	4.16	2.85	KLF9

BP_GLEAN_10012495	6	0	3.5	2.34	Zfand5
BP_GLEAN_10013248	6	0	3.11	1.69	LPL
BP_GLEAN_10013775	6	0	2.89	3.92	CIART
BP_GLEAN_10014277	6	0	3.31	2.24	Pnpla2
MSTRG.14119	6	0	2.99	1.74	--
MSTRG.14167	6	0	10.58	8.21	--
MSTRG.704	6	0	3.57	2.19	LPL
MSTRG.9159	6	0	3.61	2.29	--
MSTRG.9292	6	0	4.05	2.94	--

Supplement Table S2. GO terms enriched from 60 significantly differentially expressed genes

Class	ID	Term	Gene Number
Molecular Function	GO:0003674	nucleic acid binding transcription factor activity	4
	GO:0003674	catalytic activity	8
	GO:0003674	signal transducer activity	3
	GO:0003674	transporter activity	1
	GO:0003674	binding	19
	GO:0003674	antioxidant activity	1
	GO:0003674	molecular transducer activity	3
Cellular Component	GO:0005575	cell	12
	GO:0005575	membrane	3
	GO:0005575	macromolecular complex	1
	GO:0005575	organelle	10
	GO:0005575	organelle part	1
	GO:0005575	membrane part	3
	GO:0005575	cell part	12
Biological Process	GO:0008150	immune system process	1
	GO:0008150	metabolic process	20
	GO:0008150	cellular process	18
	GO:0008150	signaling	4
	GO:0008150	multicellular organismal process	3
	GO:0008150	developmental process	1
	GO:0008150	single-organism process	12
	GO:0008150	positive regulation of biological process	2
	GO:0008150	regulation of biological process	16
	GO:0008150	response to stimulus	6
	GO:0008150	localization	2
	GO:0008150	biological regulation	16

Supplement Table S3. List of 20 most annotated pathways to 60 significantly differentially expressed genes identified in this study enriched in KEGG analysis

KEGG_class	Pathway ID	Pathway	Qvalue	Genes
Organismal Systems	ko04710	Circadian rhythm	0.08356132	BP_GLEAN_10005816(BHLHE40); BP_GLEAN_10007289(Nr1d2); BP_GLEAN_10016005(Bhlhe40)
	ko04979	Cholesterol metabolism	0.08356132	BP_GLEAN_10013248(LPL); BP_GLEAN_10015689(SOAT1); MSTRG.704(LPL)
	ko04923	Regulation of lipolysis in adipocytes	0.09963828	BP_GLEAN_10011841(Irs2); BP_GLEAN_10014277(Pnpla2); MSTRG.9053(adrb2)
	ko04211	Longevity regulating pathway	0.12818207	BP_GLEAN_10006563(foxo1a); BP_GLEAN_10011841(Irs2); BP_GLEAN_10016747(EIF4E)
	ko04380	Osteoclast differentiation	0.14964106	BP_GLEAN_10000555(Sqstm1); BP_GLEAN_10005601(RELB); BP_GLEAN_10011616(junb)
	ko04910	Insulin signaling pathway	0.24911012	BP_GLEAN_10006563(foxo1a); BP_GLEAN_10011841(Irs2); BP_GLEAN_10016747(EIF4E)
	ko04213	Longevity regulating pathway - multiple species	0.25238026	BP_GLEAN_10006563(foxo1a); BP_GLEAN_10011841(Irs2)
	ko03320	PPAR signaling pathway	0.26763459	BP_GLEAN_10013248(LPL); MSTRG.704(LPL)
Metabolism	ko00100	Steroid biosynthesis	0.08356132	BP_GLEAN_10007279(CYP27B1); BP_GLEAN_10015689(SOAT1)
	ko00561	Glycerolipid metabolism	0.08894408	BP_GLEAN_10013248(LPL); BP_GLEAN_10014277(Pnpla2); MSTRG.704(LPL)
	ko00400	Phenylalanine, tyrosine and tryptophan biosynthesis	0.24911012	BP_GLEAN_10009421(Tat)
Cellular Processes	ko04140	Autophagy - animal	0.0900366	BP_GLEAN_10006362(Ulk2); BP_GLEAN_10008121(ZFYVE1); BP_GLEAN_10011841(Irs2); BP_GLEAN_10014551(ddit4)
	ko04210	Apoptosis	0.12818207	BP_GLEAN_10000233(diablo); BP_GLEAN_10002830(diablo); BP_GLEAN_10006143(Tnfsf10); BP_GLEAN_10011108(GADD45G)
	ko04218	Cellular senescence	0.12818207	BP_GLEAN_10000555(Sqstm1); BP_GLEAN_10005396(myc); BP_GLEAN_10006563(foxo1a); BP_GLEAN_10011108(GADD45G)
	ko04215	Apoptosis - multiple species	0.14083624	BP_GLEAN_10000233(diablo); BP_GLEAN_10002830(diablo)
Environmental Information Processing	ko04068	FoxO signaling pathway	0.09963828	BP_GLEAN_10006143(Tnfsf10); BP_GLEAN_10006563(foxo1a); BP_GLEAN_10011108(GADD45G); BP_GLEAN_10011841(Irs2)
	ko04150	mTOR signaling pathway	0.24911012	BP_GLEAN_10006362(Ulk2); BP_GLEAN_10014551(ddit4); BP_GLEAN_10016747(EIF4E)
Human Diseases	ko05216	Thyroid cancer	0.14083624	BP_GLEAN_10005396(myc); BP_GLEAN_10011108(GADD45G)
	ko04931	Insulin resistance	0.16876643	BP_GLEAN_10006563(foxo1a); BP_GLEAN_10011841(Irs2); BP_GLEAN_10015535(Trib1)
	ko05213	Endometrial cancer	0.24911012	BP_GLEAN_10005396(myc); BP_GLEAN_10011108(GADD45G)
